# Effect of Glucose or Fat Challenge on Aspirin Resistance in Diabetes

**DOI:** 10.1155/2010/820876

**Published:** 2011-01-26

**Authors:** Hussein N. Yassine, Grace Davis-Gorman, Craig S. Stump, Stephen S. Thomson, Justin Peterson, Paul F. McDonagh

**Affiliations:** ^1^Division of Endocrinology, Department of Medicine, Arizona Health Sciences Center, University of Arizona, 1656 E Mabel Street, Tucson, AZ 85724, USA; ^2^Cardiovascular and Thoracic Surgery and the Sarver Heart Center, Arizona Health Sciences Center, University of Arizona, Tucson, AZ 85724, USA; ^3^Southern Arizona VA Health Care System, Tucson, AZ 85723, USA

## Abstract

Aspirin has lower antiplatelet activity in diabetic patients. Our aim is to study the roles of acute hyperglycemia and hyperlipidemia on aspirin function in diabetic subjects with and without cardiovascular disease. Using urine thromboxane (pg/mg creatinine) and VerifyNow (Aspirin Resistance Measures-ARU), we investigated diabetic subjects during a 2-hour glucose challenge (*n* = 49) or a 4-hour fat challenge (*n* = 11). All subjects were currently taking aspirin (81 or 325 mg). After fat ingestion, urine thromboxane increased in all subjects (Mean ± SE before: after) (1209 ± 336: 1552 ± 371, *P* = .01), while we noted a trend increase in VerifyNow measures (408 ± 8: 431 ± 18, *P* = .1). The response to glucose ingestion was variable. Diabetic subjects with cardiac disease and dyslipidemia increased thromboxane (1693 ± 364: 2799 ± 513, *P* < .05) and VerifyNow (457.6 ± 22.3: 527.1 ± 25.8, *P* < .05) measures after glucose. We conclude that saturated fat ingestion increases *in vivo* thromboxane production despite aspirin therapy.

## 1. Introduction

Aspirin (ASA) is one of the most commonly used medications worldwide. There is no debate that ASA reduces mortality in secondary prevention of cardiovascular disease (CVD), but an important question is why some patients do not benefit from ASA therapy. It was suggested that diabetic patients not treated with aspirin therapy display increased platelet reactivity, and that aspirin treatment in diabetic patients is less effective than aspirin treatment in nondiabetic patients [1, 2]. This phenomenon is known as aspirin resistance (ASA-R) [3]. ASA-R is not limited to diabetes and is documented in subjects with cardiovascular and kidney disease. Despite the absence of a current gold standard to define ASA resistance [4], urine thromboxane and VerifyNow point-of-care optical aggregometry are clinically validated with cutoff values that predict worse CVD outcomes [5–7]. Urine thromboxane is a measure on *in vivo* thromboxane production. We previously demonstrated that platelets are activated after incubation with increasing glucose concentrations *in vitro* [8]. Chronic hyperglycemia and hyperlipidemia are associated with ASA-R [9], but the role of acute hyperglycemia and hyperlipidemia on ASA function *in vivo* has not been defined. In this study, we investigated the effects of acute glucose and fat ingestion on aspirin function in diabetic subjects with and without cardiovascular disease.

## 2. Materials and Methods

We studied 55 subjects with diabetes on ASA therapy for at least 2 weeks prior to testing. Subjects reported fasting to the Clinical and Translational Research Center (CATS) at 8 am. Blood was obtained on arrival and 1 hour after glucose (*n* = 49) or 4 hours after fat ingestion (*n* = 11). Five subjects did both the fat and glucose challenge on separate days. The study was approved by our Institutional Review Board, and all patients provided written informed consent prior to testing. Clinical cardiovascular disease was defined by prior coronary artery bypass surgery (CABG), percutaneous transluminal angioplasty (PTCA), or thrombotic stroke. Patients were older than 18 years of age. The study excluded subjects if they met any of the following criteria: bleeding diathesis or a history of gastrointestinal bleeding, hemorrhagic stroke, illicit drug or alcohol abuse, coagulopathy, major surgery within 6 weeks prior to study, platelet count <100,000/mm^3^, hematocrit <25%, creatinine >4 mg/dL, or current use of nonsteroidal anti-inflammatory drugs, anticoagulants, or antiplatelet drugs other than aspirin.

Blood and urine collection: after an overnight fast, blood was collected from the antecubital vein into Vacutainer tubes (Becton-Dickinson, Franklin Lakes, NJ) at 8 am to avoid a potential effect of circadian rhythm on platelet function. All patients were instructed to take their prescribed morning medications prior to blood draw. After collecting the first 2-3 mL of free flowing blood for other measurements, the tubes were filled to capacity and gently inverted 3 to 5 times to ensure complete mixing of the anticoagulant. Tubes containing 3.2% sodium citrate (Greiner Bio-One Vacuette North America, Inc., Monroe, NC) were collected for VerifyNow measurements. Urine samples were collected for measurements of 11-dh-TxB2 (Corgenix, Bloomfield, CO) and stored at −20°C until analysis. 

### 2.1. VerifyNow Aspirin Assay

VerifyNow (Accumetrics, CA) is a turbidimetric optical detection assay designed to measure platelet aggregation that is based upon the ability of activated platelets to bind to fibrinogen [6]. The cartridge contains a lyophilized preparation of human fibrinogen-coated beads, arachidonic acid, preservative, and buffer. The fibrinogen-coated beads aggregate in whole blood in proportion to the number of unblocked platelet GPIIb/IIIa receptors. The instrument reports aggregation as aspirin resistance units (ARUs). There is no significant interaction between lipemia and VerifyNow measures as per the manufacturer. The coefficient of variance was 2.5% on repeated measures within patients. The between-patient coefficient of variance was 12.5% at baseline.

### 2.2. Urinary Thromboxane (11-dh-TxB2)

AspirinWorks (Corgenix Co.) is an enzyme-linked immunosorbent assay (ELISA). In brief, assay buffer and urine were incubated with a monoclonal antibody followed by the addition of an 11-dh-TxB2-alkaline phosphate tracer. Urinary 11-dh-TxB2 concentrations were determined by measuring colorimetry at 405 nm using an ELISA reader and expressed as pg/mg Creatinine [1, 7]. The coefficient of variance was 4% on repeated measures within patients. The between-patient coefficient of variance was 14% at baseline.

### 2.3. Oral Glucose and Fat Meal

Forty-nine subjects ingested a standard glucose solution (Trutol 75, NERL Diagnostics) consisting of 75 grams of glucose. For the fat diet, 11 subjects ingested a standardized test meal [10] that consisted, per square meter of body surface area, of 175 mL of heavy whipped cream (Shamrock Farms, 99% fat). Of 700 calories per square meter, 99% (70 g) were derived from fat, <1% was derived from carbohydrates (<1 g), or from proteins (<1 g). The cholesterol content was 25 mg, and the ratio of total polyunsaturated to saturated fat was 0.06. We recorded ASA-R measures at one and two hours after glucose ingestion, and two and four hours after fat ingestion. We report the most prominent changes that we noted at one hour after glucose ingestion and four hours after lipid ingestion. We measured weight, height, waist circumference, fasting lipids, HbA1c, uric acid, serum creatinine in all subjects at baseline, and glucose or triglycerides levels after ingestion of the glucose or fat challenge, respectively.

### 2.4. Definitions

#### 2.4.1. Aspirin Resistance

Aspirin resistance was defined by previously reported criteria: ≥550 ARU by VerifyNow [5] or ≥1500 pg 11-dh-TxB2/mg creatinine during treatment with 81 mg or 325 mg of daily aspirin [7]. We defined apriori a group of responders with change of at least 10% increase (calculated as premeasure/postmeasure) in thromboxane and VN response after glucose or fat challenge.

#### 2.4.2. Statistical Analysis

Categorical variables were expressed as *n* (%) and continuous variables as mean ± SE. Nonparametric data were log transformed. Kruskal Wallis was used to analyze groups with nonparametric distribution. A one-way analysis of variance (ANOVA) for repeated measures was used to compare levels of platelet function between and within diabetic and nondiabetic patients. An unpaired *t*-test was used to compare the prevalence of aspirin resistance between groups; *P* < .05 was considered statistically significant. Linear regression was used to explain the changes in measures of ASA-R to measures of glucose or triglyceride after the challenge. All statistical calculations were performed using SPSS software (Chicago, IL).

## 3. Results

Our study subjects were mostly Caucasian, middle-aged adults. The majority of our diabetic subjects were obese and had a history of hyperlipidemia, hypertension, and were receiving medical treatment such as statins, beta-blockers, and angiotensin converting enzyme inhibitors for prevention and treatment of CVD.  Table 1 summarizes the baseline measurements.

**Figure 1:**
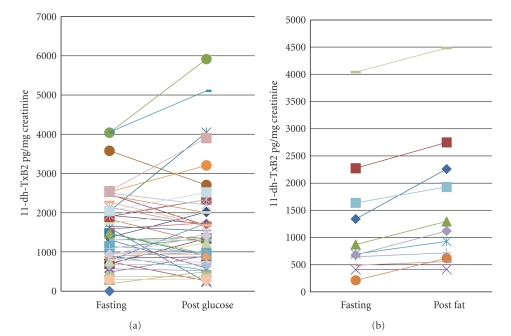
The response of urine thromboxane after glucose or fat ingestion (a). Urine thromboxane response to glucose ingestion (*n* = 49) (b). Urine thromboxane response to fat ingestion (*n* = 11). A more consistent increase in 11-dh-TxB2 was observed following the fat challenge compared to the glucose challenge. 11-dh-TxB2 = Thromboxane.

The response to glucose or fat challenge is summarized in Table 2. Urine thromboxane was significantly increased in all 11 subjects after fat ingestion (*P* = .01). Subjects demonstrated a variable response to the glucose ingestion (Figure 1). We did not find a correlation between the changes in glucose or triglyceride and the changes in 11-dh-TxB2 or VN. Within subjects that underwent the glucose challenge (*n* = 49), we had a mixed response to glucose ingestion (Figure 1(a)). We defined a group of subjects with at least 10% increase (calculated as premeasure/postmeasure) in thromboxane and VN response after glucose challenge (responders) and compared their profiles to subjects that did not change their measures of ASA-R after the glucose challenge, using an independent *t*-test. In this analysis, 13 subjects did not change their 11-dh-TxB2 measures, while 19 increased it by more than 10%. For VN measures, 14 subjects did not change their ASA-R measure, while 10 subjects had an increased response. We did not analyze the data from subjects that had a decrease in TxB2 or VN after the glucose ingestion. We log transformed the data to account for nonparametric distribution. As demonstrated in Table 3, subjects with an increased response to glucose were more aspirin resistant per 11-dh-TxB2 criteria at baseline compared to subjects where ASA-R measures did not change. Subjects with elevated 11-dh-TxB2 or VN measures in response to glucose tended to have a prior history of CVD, greater LDL cholesterol, and longer duration of diabetes. There was no correlation between the change in plasma glucose before and after glucose ingestion and measures of aspirin resistance. After glucose ingestion, two subjects were defined as aspirin resistant per the urine thromboxane criteria and three subjects per VN criteria. After fat ingestion, one subject developed ASA-R using 11-dh-TxB2 cutoff values and one subject using VN values. There was no significant correlation between the change in 11-dh-TxB2 / VN after glucose or fat ingestion.

## 4. Discussion

This is the first study to demonstrate that acute ingestion of saturated fat consistently increases *in vivo* thromboxane production, leading to a state of ASA-R. The lack of significance related to the measure of platelet function (VN) highlights that the fat load may have increased production/excretion of 11-dh-TxB2. Urinary thromboxane is not only a marker of platelet activity, but also a marker of inflammation and oxidative stress [11]. Urine thromboxane, as a global index of thromboxane synthesis, originates from other blood elements such as erythrocytes and monocytes and from renal biosynthesis [12]. We previously reported that whole blood incubation with increasing concentrations of glucose increased platelet function by the upregulation of P-selectin expression [8]. The current results suggest that the response to glucose ingestion *in vivo* is more complex. Subjects with chronic hyperglycemia, as assessed by the duration of diabetes, dyslipidemia, and prior CVD, are likely to be ASA-R in response to a high glucose load. The state of increased thromboxane production and platelet activity after an acute fat or glucose load may be a mechanism explaining the role of a heavy meal as a trigger for acute myocardial infarction [13, 14].

Although there is no gold standard for defining ASA-R, definitions based on urine 11-dh-TxB2 and VN assays have cutoff values that are clinically validated to predict worse CVD outcomes. In a subanalysis of the Heart Outcomes Prevention Evaluation (HOPE) study, the odds, over a 5-year period, of the composite outcome of myocardial infarction, stroke, or CVD death in 976 ASA-treated patients (488 who suffered myocardial infarction, stroke, or CVD death and 488 sex- and age-matched controls) increased with each increasing quartile urine thromboxane levels [7]. The antiplatelet effect of aspirin measured by VerifyNow correlated with adverse clinical events in stable patients with CAD and is independent of known risk factors including diabetes and prior MI [5]. The prevalence of ASA-R in our study was 36% by urine 11-dh-TxB2, and 18% by VN measures (Table 1). In comparison, the prevalence of ASA resistance of diabetic subjects on 81 mg of ASA in the ASPECT study was 37% by urine 11-dh-TxB2, and 13% for VN [1]. In another study [15] of subjects with CVD but not diabetes, ASA-R prevalence was 23% by 11-dh-TxB2, and 7% by VN.

Possible mechanism(s) for the reduced ability of ASA to sufficiently protect against intravascular occlusions in diabetes mellitus (DM) patients compliant with ASA therapy include chronic hyperglycemia, hyperlipidemia, and suboptimal ASA dosing, particularly in relation to altered platelet turnover in diabetic patients [16, 17]. The activating role of chronic hyperlipidemia on platelet function was demonstrated both *in vivo* [18] and *in vitro* [19]. Friend et al. [18] found that platelet responsiveness to aspirin is reduced in patients with chronic hyperlipidemia. Davi et al. [20] observed enhanced *in vivo* formation of the F2-isoprostane 8-epi-PGF2a in chronic hyperlipidemia that correlated with enhanced urine 11-dh-TxB2 production. This provides an aspirin-insensitive mechanism possibly linking lipid peroxidation to amplification of platelet activation. Moreover, platelet responsiveness to ASA improves after statin therapy [21]. Alternatively, oxidized LDL and oxidized choline glycerophospholipids induce platelet activation and thrombosis in fat fed mice through platelet CD36 [19]. The molecular mechanisms explaining platelet response to acute glucose loads might be more complex. We suggest that rather than acute hyperglycemia per se, it is the mishandling of hyperglycemia and resulting libration of oxygen-free radicals [22] in subjects with chronic diabetes, dyslipidemia, and prior cardiovascular disease leading to increased platelet activation and thromboxane production. 

The limitations of this study are the small sample size, the absence of a control group, and a CVD cohort without diabetes. The acute response to glucose or fat ingestion may not reflect the chronic condition. Although we noted a trend increase in VerifyNow after fat ingestion, the lack of a significant change is possibly related to our small cohort size. The greater variability in the prechallenge 11-dh-TxB2 between the glucose and fat groups is likely related to the small size of the fat challenge group.

## 5. Conclusion

In summary, our study indicates that saturated fat in the diet increases *in vivo* thromboxane production. This highlights the importance of low dietary saturated fat intake and aggressive lipid management in diabetic patients on ASA therapy.

##  Conflict of Interests 

The authors have no conflict of interests to disclose. 

## Figures and Tables

**Table 1:** Baseline measures.

Patient characteristic	Diabetics (*n* = 56)
Gender (M/F)	28/28
ASA dose (81 mg/325 mg)	48/8
Prior CVD	8/58 (14%)
Smoking, *n* (%)	7/49 (14%)
Age (years)	59 ± 1.5
BMI (kg/m^2^)	37 ± 3
Medication (%)	
ACE inhibitors or ARB	36%
Statin	51%
Insulin	43%
Waist circumference (cm)	111 ± 2
Systolic BP (mmHg)	130 ± 2
Diastolic BP (mmHg)	78 ± 1.2
11-dh-TxB2 (pg/mg creatinine)	1465 ± 125
ASA-S (%)	64%
VerifyNow (ARU)	455 ± 12
ASA-S (%)	82%
LDL (mg/dL)	105 ± 5
HDL (mg/dL)	47 ± 2
TG (mg/dL)	210 ± 26
Creatinine (mg/dL)	0.8 ± 0.04
HbA1c %	8.5 ± 0.3
Fasting Glucose (mg/dL)	169 ± 11
Duration of diabetes (years)	10.5 ± 1

Values are presented in means ± SE.

11-dh-TxB2: Urine thromboxane, ARU: Aspirin resistance units, TG:  Triglyceride, LDL: Low density lipoproteins, HDL: High density lipoprotein, ASA-S: Aspirin sensitive, ARB: Angiotensin receptor blocker, ACE: Angiotensin converting enzyme.

**Table 2:** Response to glucose or fat challenge.

	Glucose challenge (*n* = 49)				Fat challenge (*n* = 11)		
	Pre	Post	*P* value		Pre	Post	*P* value
Glucose(mg/dL)	169 ± 11	289 ± 16	<.001	Triglyceride (mg/dL)	210 ± 26	369 ± 118	<.001
11-dh-TxB2 (pg/mg creatinine)	1538 ± 131	1806 ± 247	.16	11-dh-TxB2 (pg/mg creatinine)	1209 ± 336	1552 ± 371	.01
VerifyNow (ARU)	461 ± 12	453 ± 13	.4	VerifyNow (ARU)	408 ± 8	431 ± 18	.1

Values are presented in means ± SE.

**Table 3:** Response to glucose ingestion.

	11-dh-TxB2 (pg/mg of creatinine)		VN (ARU)	
	Nonresponders (*n* = 13)	Responders (*n* = 19)	Nonresponders (*n* = 14)	Responders (*n* = 10)
Pre-Glucose % of ASA-R	942 ± 117	1693 ± 364^†^	430.4 ± 18.5	457.6 ± 22.3
	15	55	7	10
Post-Glucose % ASA-R	954.1 ± 122	2799 ± 513^†‡^	428.5 ± 18.1	527.1 ± 25.8^‡^
	15	63	7	33
CVD (*n*)	1	4^†^	0	4^†^
Duration of diabetes	6.3 ± 1.7	8.4 ± 1.7	4.9 ± 1.4	11.1 ± 2.3^†^
LDL (mg/dL)	102 ± 8.6	126 ± 8.3^†^	105 ± 9.4	121 ± 11.6

Responders were defined by more than 10% increase in ASA-R measures after glucose ingestion. ASA-R: aspirin resistant, ASA-S: aspirin sensitive, CVD: cardiovascular disease.

Data are presented as means ± SE. The following group comparisons denote *P* < .05. ^†^Non-responders: Responders, ^‡^Pre: Post.
